# Supplementary material for: The Association between Childhood Immunization and Gender Inequality: A Multi-Country Ecological Analysis of Zero-Dose DTP Prevalence and DTP3 Immunization Coverage
Source: Vaccines (Basel). 2022 Jun 27;10(7):1032. doi: 10.3390/vaccines10071032 (PMC9317382; doi:10.3390/vaccines10071032)
Supplement: Supplementary file 1 [file vaccines-10-01032-s001.zip › vaccines-1734563-supplementary.pdf]

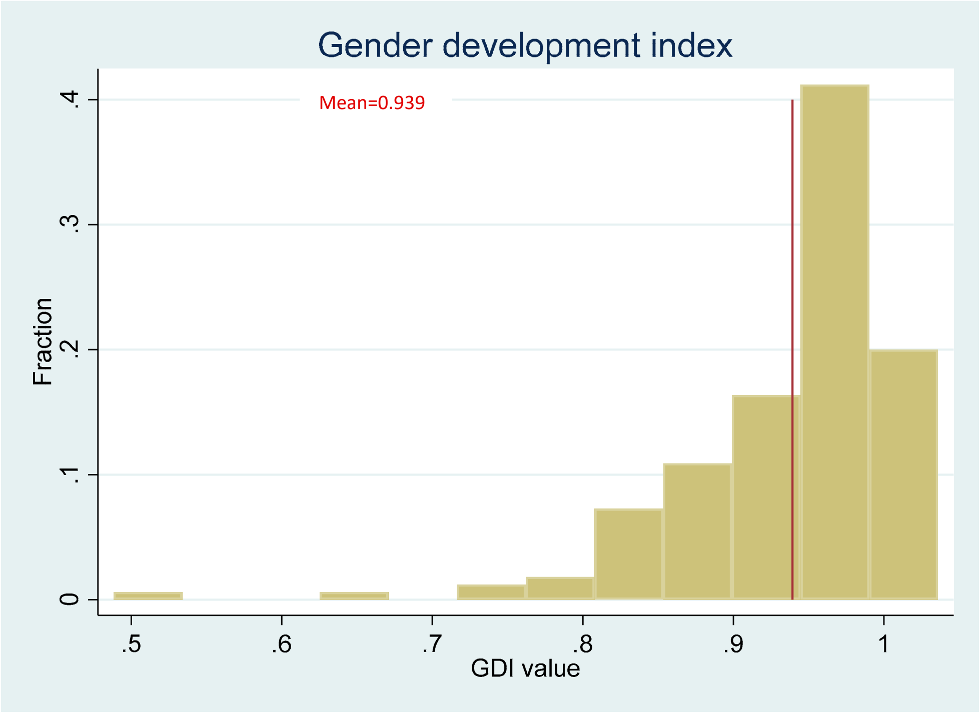

Supplementary Figure S1. GDI distribution (165 countries, 2019).

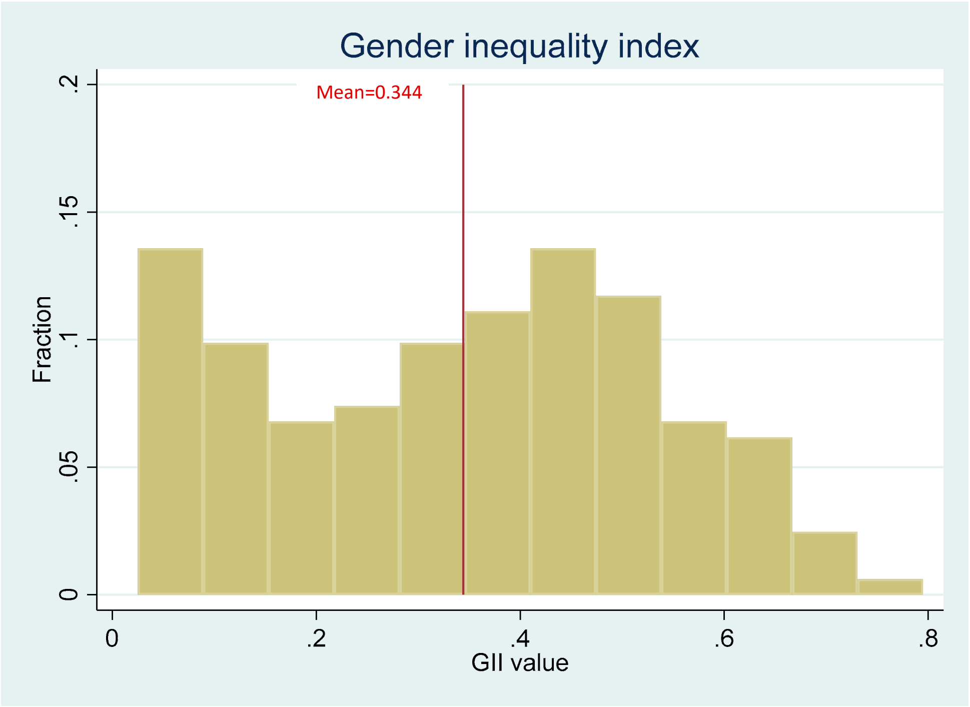

Supplementary Figure S2. GII distribution (162 countries, 2019).

Supplementary Table S1. Determinants of zero-dose DPT in one-year old children, GDI (2010-2019).

VARIABLES	(1)<br>Zerodpt-<br>Model 1	(2)<br>Zerodpt-<br>Model 2	(3)<br>Zerodpt-<br>Model 3	(4)<br>Zerodpt-<br>Model 4
**High gender inequality (favoring men)**	**1.295 *****<br>**(0.191)**	**0.476 *****<br>**(0.181)**	**0.445 ****<br>**(0.176)**	**0.524 *****<br>**(0.202)**
Annual population growth (%)		-0.129 *<br>(0.0694)	-0.223 ***<br>(0.0487)	-0.172 **<br>(0.0822)
Population under 15 years (%)		0.0149<br>(0.0265)	0.0260<br>(0.0237)	0.0426<br>(0.0280)
Urban population (%)		0.00764<br>(0.00530)	0.0101*<br>(0.00520)	0.00927<br>(0.00635)
Human development index		-3.880 **<br>(1.515)		
Health index		0.141<br>(1.408)		
Economic index		-1.156<br>(0.948)		
Education index			-4.049 **<br>(1.622)	
Log GDP per capita PPP				0.0182<br>(0.286)
Mean years schooling pop 25+				0.00563<br>(0.0454)
Log current health expenditure pc PPP				-0.329<br>(0.202)
Constant	-3.095 ***<br>(0.124)	-0.0844<br>(1.998)	-1.218<br>(1.671)	-4.057 **<br>(1.722)
Observations	1628	1610	1618	1401

Dependent variable is proportion of one-year old children that have not received any dose of the DTP vaccine. All models were estimated using fractional outcome regression. Fractional response estimators fit models on continuous zero to one data using logit (STATA command fracreg logit). Data used for years 2011-2019. All models include year fixed effects. Robust standard errors in parenthesis. Excluded base category for GDI is High gender inequality (favoring men).

* $p < 0.10$; ** $p < 0.05$; *** $p < 0.01$.

Supplementary Table S2. Determinants of DPT3 immunization coverage in one-year old children, GDI (2010-2019).

VARIABLES	(1)<br>dpt3–<br>Model 1	(2)<br>dpt3–<br>Model 2	(3)<br>dpt3–<br>Model 3	(4)<br>dpt3–<br>Model 4
**High gender inequality (favoring men)**	**-1.281 *****	**-0.462 *****	**-0.449 *****	**-0.542 *****
	**(0.178)**	**(0.160)**	**(0.162)**	**(0.184)**
Annual population growth (%)		0.169 **	0.247 ***	0.188 *
		(0.0736)	(0.0563)	(0.0965)
Population under 15 years (%)		-0.0167	-0.0266	-0.0452 *
		(0.0238)	(0.0211)	(0.0242)
Urban population (%)		-0.0115 **	-0.0135 ***	-0.0104 *
		(0.00496)	(0.00473)	(0.00600)
Health index		3.428 ***		
		(1.317)		
Economic index		0.449		
		(1.199)		
Education index		1.344 *		
		(0.790)		
Human development index			4.459 ***	
			(1.479)	
Log GDP per capita PPP				0.160
				(0.231)
Mean years schooling pop 25+				0.00619
				(0.0428)
Log current health expenditure pc PPP				0.133
				(0.143)
Constant	2.506 ***	-0.474	0.511	3.036 **
	(0.0988)	(1.800)	(1.487)	(1.426)
Observations	1628	1610	1618	1401

Dependent variable is proportion of one-year old children that have 3 doses of the DTP vaccine. All models were estimated using fractional outcome regression. Fractional response estimators fit models on continuous zero to one data using logit (STATA command fracreg logit). Data used for years 2011-2019. All models include year fixed effects. Robust standard errors in parenthesis. Excluded base category for GDI is High gender inequality (favoring men). * $p < 0.10$; ** $p < 0.05$; *** $p < 0.01$.

Supplementary Table S3. Average partial effects of GDI on the predicted value of zero-dose DTP and DTP3 coverage (2010-2019).

	Model 1<br>(no controls)	Model 2	Model 3	Model 4
Zero-dose children				
High gender inequality	0.106 ***	0.031 **	0.028 **	0.033 **
95% CI	0.06-0.15	0.01-0.06	0.01-0.05	0.01-0.06
DTP3 coverage				
High gender inequality	-0.158 ***	-0.046 ***	0.044 **	0.054 ***
95% CI	-0.22--0.10	-0.08--0.01	-0.08--0.01	-0.09--0.01

* $p< 0.10$; ** $p < 0.05$; *** $p< 0.01$.

Supplementary Table S4. Determinants of zero-dose DPT in one-year old children, GII (2010-2019).

VARIABLES	(1)<br>Zerodpt-<br>Model 1	(2)<br>Zerodpt-<br>Model 2	(3)<br>Zerodpt-<br>Model 3	(4)<br>Zerodpt-<br>Model 4
**Gender inequality index**	**3.666** ***	**3.077** ***	**2.990** ***	**3.290** ***
	**(0.424)**	**(0.941)**	**(0.941)**	**(0.973)**
Annual population growth (%)		-0.0627	-0.193 **	-0.0585
		(0.0621)	(0.0824)	(0.0616)
Population under 15 years (%)		-0.625	0.765	-0.688
		(2.740)	(2.440)	(2.834)
Urban population (%)		0.00225	0.00529	0.00499
		(0.00668)	(0.00639)	(0.00694)
Health index		-2.087		
		(1.422)		
Economic index		0.0314		
		(1.556)		
Education index		-0.142		
		(1.183)		
Human development index			-1.901	
			(1.810)	
Log GDP per capita PPP				-0.0193
				(0.301)
Mean years schooling pop 25+				0.0420
				(0.0527)
Log current health expenditure pc PPP				-0.317
				(0.196)
Constant	-4.365 ***	-2.317	-3.035 *	-3.940 ***
	(0.226)	(2.018)	(1.736)	(1.513)
Observations	1559	1541	1559	1343

Dependent variable is proportion of one-year old children that have not received any dose of the DTP vaccine. All models were estimated using fractional outcome regression. Fractional response estimators fit models on continuous zero to one data using logit (STATA command fracreg logit). Data used for years 2011-2019. All models include year fixed effects. Robust standard errors in parenthesis. * $p < 0.1$; ** $p < 0.05$; *** $p < 0.01$.

Supplementary Table S5. Determinants of DPT3 coverage in one-year old children, GII (2010-2019).

VARIABLES	(1)<br>dpt3–<br>Model 1	(2)<br>dpt3–<br>Model 2	(3)<br>dpt3–<br>Model 3	(4)<br>dpt3–<br>Model 4
**Gender inequality index**	**-3.643 *****	**-2.876 *****	**-2.833 *****	**-3.422 *****
	**(0.382)**	**(0.836)**	**(0.833)**	**(0.874)**
Annual population growth (%)		0.130 *	0.231 ***	0.0901
		(0.0687)	(0.0780)	(0.0683)
Population under 15 years (%)		-0.380	-1.417	-0.238
		(2.580)	(2.307)	(2.539)
Urban population (%)		-0.00555	-0.00805	-0.00553
		(0.00612)	(0.00584)	(0.00637)
Health index		1.751		
		(1.294)		
Economic index		0.124		
		(1.320)		
Education index		0.428		
		(1.017)		
Human development index			2.104	
			(1.744)	
Log GDP per capita PPP				0.141
				(0.236)
Mean years schooling pop 25+				-0.0327
				(0.0485)
Log current health expenditure pc PPP				0.108
				(0.125)
Constant	3.759 ***	2.008	2.530	3.387 **
	(0.190)	(1.915)	(1.639)	(1.325)
Observations	1559	1541	1559	1343

Dependent variable is proportion of one-year old children that have 3 doses of the DTP vaccine. All models were estimated using fractional outcome regression. Fractional response estimators fit models on continuous zero to one data using logit (STATA command fracreg logit). Data used for years 2011-2019. All models include year fixed effects. Robust standard errors in parenthesis. * $p < 0.1$; ** $p < 0.05$; *** $p < 0.01$.
